# Impact of metronomic trabectedin combined with low-dose cyclophosphamide on sarcoma microenvironment and correlation with clinical outcome: results from the TARMIC study

**DOI:** 10.1186/s12943-024-01942-y

**Published:** 2024-02-19

**Authors:** Cheng-Ming Sun, Maud Toulmonde, Mariella Spalato-Ceruso, Florent Peyraud, Alban Bessede, Michèle Kind, Sophie Cousin, Xavier Buy, Jean Palussiere, Antoine Bougouin, Catherine Sautès-Fridman, Hervé Wolf Fridman, Marina Pulido, Antoine Italiano

**Affiliations:** 1 Centre de Recherche des Cordeliers, Paris, France; 2 Department of Medicine, Institut Bergonié, Bordeaux, France; 3 Explicyte, Bordeaux, France; 4 Department of Imaging, Institut Bergonié, Bordeaux, France; 5 Clinical and Epidemiology Department, Institut Bergonié, Bordeaux, France; 6 Faculty of Medicine, University of Bordeaux, Bordeaux, France

## Abstract

Soft tissue sarcomas (STS) are diverse mesenchymal tumors with few therapeutic options in advanced stages. Trabectedin has global approval for treating STS patients resistant to anthracycline-based regimens. Recent pre-clinical data suggest that trabectedin’s antitumor activity extends beyond tumor cells to influencing the tumor microenvironment (TME), especially affecting tumor-associated macrophages and their pro-tumoral functions. We present the phase I/II results evaluating a combination of metronomic trabectedin and low-dose cyclophosphamide on the TME in patients with advanced sarcomas. 50 patients participated: 20 in phase I and 30 in phase II. Changes in the TME were assessed in 28 patients using sequential tumor samples at baseline and day two of the cycle. Treatment notably decreased CD68 + CD163 + macrophages in biopsies from tumor lesions compared to pre-treatment samples in 9 of the 28 patients after 4 weeks. Baseline CD8 + T cell presence increased in 11 of these patients. In summary, up to 57% of patients exhibited a positive immunological response marked by reduced M2 macrophages or increased CD8 + T cells post-treatment. This positive shift in the TME correlated with improved clinical benefit and progression-free survival. This study offers the first prospective evidence of trabectedin’s immunological effect in advanced STS patients, highlighting a relationship between TME modulation and patient outcomes.

This study was registered with ClinicalTrial.gov, number NCT02406781.

**Supplementary Information:**

The online version contains supplementary material available at 10.1186/s12943-024-01942-y.

## Introduction

Metronomic chemotherapy (MTC) entails the consistent and frequent administration of lower doses of chemotherapeutic drugs with limited drug-free intervals [1]. This approach has been recognized for its impact on the immune cells within the tumor microenvironment, specifically reducing the abundance of immune cells that foster tumor growth, such as regulatory T cells and myeloid-derived suppressor cells [2]. Among MTC regimens, low-dose cyclophosphamide stands out as the most frequently employed in the clinical setting, demonstrating both antiangiogenic effects and immunomodulatory actions [3, 4]. In the realm of sarcomas, low-dose cyclophosphamide has exhibited clinical benefits in retrospective and prospective studies [4, 5].

Concurrently, trabectedin, a marine-derived antineoplastic agent approved in Europe, the United States, and Asia for managing advanced soft-tissue sarcomas, showcases a distinctive mechanism of action [6, 7]. Binding to the minor groove of DNA, trabectedin induces DNA strand breaks, disrupting the transcription process. Beyond its direct antitumor effects, trabectedin also influences the tumor microenvironment, potentially modulating immune response and angiogenesis [8].

This correspondence presents the outcomes of the TARMIC study, which aimed to evaluate the safety, efficacy, and immunologic effects of weekly intravenous administration of trabectedin in combination with oral low-dose cyclophosphamide in patients with advanced soft-tissue sarcomas (STS).

## Methods

### Trial design and oversight

TARMIC is a phase 1/2 study enrolling patients with advanced sarcomas at Institut Bergonié (Bordeaux, France). The phase 1 part of the study followed a 3 + 3 classical design with four doses levels of weekly trabectedin: 0.3 mg/m², 0.4 mg/m², 0.5 mg/m² and 0.6 mg/m². The phase 2 part of the study was based on Simon’s optimal two-stage design. The clinical trial was conducted in accordance with the Declaration of Helsinki and Good Clinical Practices. All patients provided written informed consent. For the phase 2 part, confirmation of disease progression based on central review of two imaging obtained at less than a 6-months interval was mandatory at inclusion. As required by the French regulation, the protocol was centrally approved by a central IRB (the Comité de Protection des Personnes Sud-Ouest et Outre Mer III, Bordeaux, France) which reviewed the appropriateness of the clinical trial protocol as well as the risks and benefits to study participants. All patients provided written informed consent. Safety and efficacy data were reviewed by an independent data monitoring committee.

### Patients

Main eligibility criteria included: Age ≥ 18 years; Eastern Cooperative Oncology Group (ECOG) performance status ≤ 1; Histological confirmation of soft-tissue sarcoma by central review; documented progression according to RECIST criteria [9]. For the phase 2 part, progression on the last line of treatment should be confirmed by central review with two radiological assessments identical (CT scans or MRI) obtained at less than 6 months interval within the 12 months before inclusion, except for patients with metastatic disease diagnosed less than 6 months before inclusion; locally advanced or metastatic disease; and adequate hematological, renal, metabolic, and hepatic functions. Full list of eligibility criteria is available in the protocol.

### Study treatment

All patients received trabectedin weekly for three consecutive weeks (days 1, 8 and 15) every 4 weeks and cyclophosphamide 50 mg BID 1week on, 1 week off. Four dose levels (DL) of trabectedin were assessed during the dose escalation phase (DL1: 0.3 mg/m², DL2: 0.4 mg/m², DL3: 0.5 mg/m², DL4: 0.6 mg/m²). Patients discontinued treatment if one of the following occurred: the patient made the decision to withdraw or there was unacceptable toxicity, disease progression according to RECIST 1.1 criteria [9], undercurrent illness, or changes in patient condition preventing further treatment at the discretion of the investigator.

### Response assessment and toxicity

Fist tumor assessment was performed at week 6 and every 6 weeks thereafter. Response was determined according to RECIST 1.1 guidelines [9] after blinded central imaging review. Toxicities were assessed continuously according to Common Terminology Criteria for Adverse Events v.4.0.

### Tumor biopsies and immunohistochemistry (IHC) and multiplex immunofluorescence (IF) assay

Patients underwent tumor biopsies both at baseline and on cycle 2, Day 1, allowing for a comprehensive evaluation of the chemotherapy’s impact on the tumor microenvironment. Formalin-fixed paraffin-embedded (FFPE) tissues were precisely sectioned to 3–4 μm thickness. Antigen retrieval was conducted on a PT-link (Dako) using EnVision FLEX Target Retrieval Solutions, either at High pH (Dako, K8004) or Low pH (Dako, K8005). To minimize interference, endogenous peroxidase activity and non-specific Fc receptor binding were blocked with 3% H2O2 (Gifrer, 10,603,051) and Protein Block (Dako, X0909), respectively. Primary, secondary antibodies, and tertiary reagents utilized for Immunohistochemistry (IHC) and Immunofluorescence (IF) are detailed in supplementary Table 1. All image analyses were conducted by a single reviewer (AB), excluding necrotic and serous areas from evaluation. For IHC, FFPE sections were stained with CD8 (M7103, Agilent), followed by chromogenic detection using DAB (Agilent, K346811-2), and counterstaining of nuclei with hematoxylin (Dako, S3301). Following mounting with Glycergel (Agilent, C056330-2) or EcoMount (Biocare Medical, EM897L), slides were scanned with a NanoZoomer (Hamamatsu). CD163-CD68-2plex staining was manually performed using a tyramide system amplification (TSA) and a conventional fluorescent-dye conjugated secondary antibody system (1:100). Nuclei were stained with DAPI Solution (Thermofisher, 62,248) at 2 µg/ml for 10 min. Post-mounting with ProLongTM Gold Antifade Mountant (Thermofisher, P36934), slides were scanned at 20X magnification using a Zeiss Axio scan Z1 device. Positive cell density was quantified using Halo10 software (Indica labs) employing fitting counting algorithms.

### Statistical analysis

The primary endpoint for the phase 1 part of the study was the definition of the recommended phase 2 dose of weekly trabectedin. The primary endpoint for the phase 2 part of the study was 6-month non-progression according to RECIST 1.1 criteria, and a Simon’s two-stage design was used. This was computed as the number of patients alive and progression-free at six months, divided by the number of patients included in the population of patients evaluable for efficacy. To be considered in the population of patients evaluable for efficacy, patients had to meet the eligibility criteria and receive at least one infusion of trabectedin and one dose of CP. As such, patients of the population of patients evaluable for efficacy who died before 6 months were counted in the denominator, but not in the numerator. To distinguish a favorable true non-progression rate of 40% from a null rate of 20% (with 80% power and 5% type I error), 43 eligible and assessable patients were needed. Using 3-month or 6-month PFSs as the principal endpoints for phase 2 trials enrolling patients with STS is an international recommendation from the Sarcoma Tumor Group of the EORTC. Based on these recommendations, a 6-month PFS rate of > or = 30% is considered as a reference value to suggest drug activity in the 1st line setting; for second-line therapy, a 3-month PFS rate of > or = 40% would suggest a drug activity, and < or = 20% would suggest inactivity. Following inclusion of the first 13 assessable patients, accrual could continue for a total of 43 patients if at least four non-progressions were observed. At the end of recruitment, at least thirteen non-progressions were needed to conclude that the investigational treatment had a meaningful effect. Secondary endpoints included the best overall response according to RECIST 1.1 criteria, 1-year PFS, 1-year OS, safety, and correlations with the immunological characteristics of the tumors. PFS was defined as the time from the start of treatment to the time of progression or death (from any cause). OS was defined as the time from the start of treatment to death (from any cause) or the last patient contact. Patients who were alive and progression-free were censored at the date of last follow-up. All enrolled patients who received at least one dose of one of the investigational drugs were eligible for safety analyses. To be assessed for the primary efficacy endpoint, a subject had to meet the eligibility criteria and receive at least one dose of CP and one infusion of trabectedin. Descriptive statistics were used to characterize patients at study entry and report toxicities.

Survival rates were estimated using the Kaplan–Meier method. Patients were classified as “high” or “low” for the different immune cell subsets based on an optimal cut-point value computed using the “survminer” R package (https://cran.r-project.org/web/packages/survminer/index.html). Differences between groups were evaluated using the Wilcoxon-Mann-Whitney test for continuous variables. All comparisons performed for the translational analyzes were exploratory and hypothesis generating.

### Data availability

The datasets that support the findings of this study are not publicly available due to information that could compromise research participant consent. According to French/European regulations, any re-use of the data must be approved by the appropriate ethics committee. Individual participant data that underlie the results reported in this article can be shared upon request to the corresponding author (AI). Proposals may be submitted up to 36 months following article publication.

## Results

Between December 8, 2015 and August 3, 2019, 50 patients were included in the study: 20 patients in the dose escalation phase, 30 patients in the phase 2 part (Supplementary Fig. 1). Their baseline characteristics are listed in Supplementary Tables 2 and 3.

In the dose escalation phase, 3 patients were treated at dose level 1, 3 patients were treated at dose level 2, 7 patients at dose level 3, 7 patients at dose level 4. 3 DLT occurred in 2 patients: 1 patient experienced grade 4 CPK increase and in 1 patient Grade 4 gamma-glutamyl transferase (GGT) as well as febrile neutropenia. Adverse events related to treatment are described in Supplementary Table 4. The recommended dose to administer in the phase II part was the dose level 3: 0.50 mg/m². 16 patients were evaluable for efficacy. Two patients were progression-free at 6 months, and the 6-month non-progression rate was 12.5% (95% CI: 1.5–38.3). The best response was stable disease for 10 patients [10 leiomyosarcoma] and progressive disease for 6 patients [2 leiomyosarcomas, 1 dedifferentiated liposarcoma, 1 myxoid liposarcoma, 1 synovial sarcoma, 1 undifferentiated pleomorphic sarcoma]. Median PFS was 3.5 months (95% CI: 1.8–3.7 months). The 6-month and 1-year PFS rates were 18.8% (95% CI: 4.6–40.2%) and 6.3% (95% CI: 0.4–24.7%), respectively. Additionally, the median overall survival (OS) was 14.9 months (95% CI: 6.9 months – 28.7 months).

Of the 30 enrolled in the phase 2 part, 24 were eligible and assessable for efficacy (Supplementary Fig. 1). After a median follow-up of 12.0 months (95% CI: 11.8–12.0), three patients were (10%) still receiving treatment. Discontinuation was related to disease progression in 24 cases (73%) and investigator decision for three patients (15.5%) (Supplementary Fig. 1). Three patients were progression-free at 6 months, and the 6-month non-progression rate was 12.5% (95% CI: 2.7–32.4), indicating that the first endpoint of the study was not reached. Five patients (29.4%) had tumor shrinkage resulting in partial response in 2 case (5.9%) and stable disease in 3 cases (23.5%) (Supplementary Fig. 2A). The best response was partial response for two patients [1 undifferentiated pleomorphic sarcoma, 1 leiomyosarcoma], stable disease for 9 patients [1 dedifferentiated liposarcoma, 1 myxoid liposarcoma, 3 leiomyosarcomas, 2 synovial sarcoma, 2 undifferentiated pleomorphic sarcoma] and progressive disease for 14 patients [3 dedifferentiated liposarcoma, 2 leiomyosarcoma, 1 myxofibrosarcoma, 3 synovial sarcoma, 4 undifferentiated pleomorphic sarcoma]. Median PFS was 1.9 months (95% CI: 1.7–4.4 months) (Supplementary Fig. 2B). The 6-month and 1-year PFS rates were 12.5% (95% CI: 3.1–28.7%) and 8.3% (95% CI: 1.4–23.3%), respectively. Additionally, the median overall survival (OS) was 12.0 months (95% CI: 5.8 months – NA) (Supplementary Fig. 2C). The safety analysis was reported in Supplementary Table 5.

**Fig. 1:**
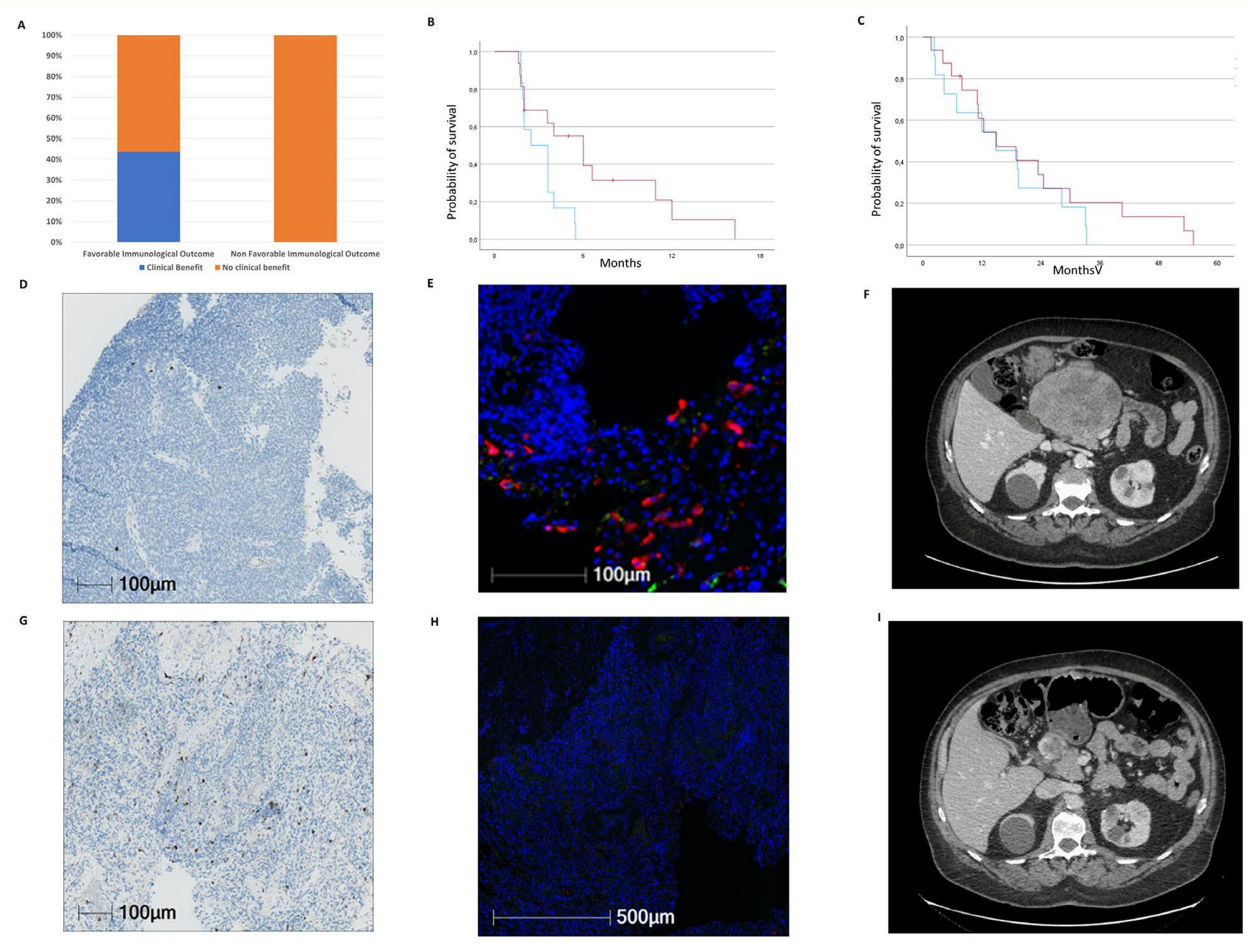
Histogram of clinical benefit (objective response plus stable disease > 6 months) Waterfall plots of tumor response (**A**), Kaplan-Meier curves of progression-free (**B**), overall survival (**C**) and histological (**D,E,G,H**)/imaging assessment (**F,I**) according to immunological outcome. Only patients with accessible tumor material for immunological analysis are included (*n* = 28). Changes in tumor size were centrally assessed by blinded independent review according to Response Evaluation Criteria in Solid Tumors (RECIST) 1.1. Blue curve: no favorable immunological outcome, red curve: favorable immunological outcome. (**D,G**) Density of CD8 + T cells within the tumor on pre-treatment (**D**) and on-treatment (**G**) samples in a patient with undifferentiated pleomorphic sarcoma enrolled in the phase 2 part. (**E**,**H**) Density of CD68+(red)CD163+(green) macrophages within the tumor on pre-treatment (**E**) and on-treatment samples (**H**) in a patient with undifferentiated pleomorphic sarcoma. Long lasting partial response (**F,I**) in a patient with undifferentiated pleomorphic sarcoma

We have evaluated the ability of the TARMIC regimen to alter the tumor microenvironment of STS in sequential tumor samples obtained at baseline and at cycle 2 Day 1 treatment in 28 patients enrolled in the dose escalation part (dose levels 3 and 4) and in the phase 2 part. The treatment induced reduction of CD68 + CD163 + macrophages in on-treatment biopsies from tumor lesions compared to matched biopsies taken from the same lesion prior to treatment in 9 patients analyzed after 4 weeks of therapy (2 leiomyosarcomas, 1 dedifferentiated liposarcoma, 1 myxofibrosarcoma, 3 undifferentiated pleiomorphic sarcomas, 2 synovial sarcomas) (Supplementary Fig. 3A). The baseline CD8 + T cell infiltrate increased in 11 patients (5 leiomyosarcomas, 1 dedifferentiated liposarcoma, 1 round cell liposarcoma, 1 myxofibrosarcoma, 2 undifferentiated pleomorphic sarcomas, 1 synovial sarcoma) (Supplementary Fig. 3B). Overall, 16 patients (6 leiomyosarcomas, 2 dedifferentiated liposarcomas, 1 round cell liposarcoma, 1 myxofibrosarcoma, 2 synovial sarcomas, 4 undifferentiated pleiomorphic sarcomas) had a favorable immunological outcome characterized by a decrease in M2 macrophage or an increase in CD8 + T cells upon treatment. This favorable alteration of the microenvironment was associated with a significantly improved clinical benefit rate (objective response plus stable disease lasting at least 6 months): 46.7% vs. 0%, *p* = 0.007), PFS: 6 months (95% CI 2.7–9.2) vs. 2.4 months (95% CI 1.1–3.8), *p* = 0.014 (Fig. 1). No statistically significant change in terms of overall survival was observed: 14.9 months (95% CI 5.3–24.5) vs. 11.9 months (95% CI 0-25.5), *p* = 0.28 (Fig. 1). Figure 1 (D-I) illustrates the density of CD8 + T cells and M2 macrophages within the tumor in pre-treatment and on-treatment samples from a patient with undifferentiated pleomorphic sarcoma enrolled in the phase 2 part, exhibiting a long-lasting partial response (> 12 months). Among the 28 patients with accessible tumor material for microenvironment analysis, 22 underwent further systemic treatments following TARMIC discontinuation (median number of subsequent lines of treatment: 2, range: 1–6).

## Discussion

Chemotherapy is still the mainstay treatment for patients with advanced sarcomas. However, its effects is not limited to the tumor cells alone. Trabectedin is approved in Europe for the management of patients with advanced STS refractory to anthracycline-based chemotherapy regimen. One of the most studied impacts of trabectedin is on tumor associated macrophages [8]. Trabectedin has been shown to selectively deplete pro-tumorigenic tumor associated macrophages from the TME and to increase T cells in in vivo models [8, 10]. Our study shows that such impact on TME is observed in about 57% of patients and associated with improved PFS: 6 months (95% CI 2.7–9.2) vs. 2.4 months (95% CI 1.1–3.8), *p* = 0.014. However, we did not observe any impact on overall survival probably due to the subsequent therapies the patients received. Of note, it is important to consider that the combination of trabectedin with low-dose cyclophosphamide might have exerted a synergistic impact on the tumor microenvironment. Additionally, the potential immunosuppressive impact of steroid premedication used in conjunction with trabectedin warrants further investigation. In this context, lurbinectedin, an analogue known to possess immunomodulatory properties similar to trabectedin but without the need for steroid premedication, emerges as a promising candidate for future studies aimed at modulating the sarcoma microenvironment [11]. Future studies should be adequately powered to account for the heterogeneity of STS, enabling a more detailed exploration of trabectedin’s impact on specific histological subtypes.Trabectedin’s ability to modulate the tumor microenvironment in STS positions it as an attractive candidate for combination with immune checkpoint inhibitors. Preclinical studies have indeed demonstrated synergistic effects when trabectedin is paired with anti-PD-1 antibodies [10]. However, investigations into the co-administration of trabectedin and immune checkpoint inhibitors in advanced STS patients yielded only modest clinical benefits [12, 13]. A potential explanation is that in these studies, the combination was administered upfront. Given that our data reveals trabectedin influences the tumor microenvironment in only a select group of patients, a sequential approach might be more appropriate. This would involve administering anti-PD-1 specifically to patients who demonstrate a favorable immunological response to trabectedin.

## Conclusions

In conclusion, the TARMIC study underscores the potential of trabectedin combined with low-dose cyclophosphamide in modifying the tumor microenvironment of advanced soft-tissue sarcomas. This regimen demonstrated a capacity to enhance progression-free survival, particularly in patients exhibiting favorable immunological changes. While the impact on overall survival remains unclear, these findings encourage further exploration of this combination, particularly in tandem with immune checkpoint inhibitors, to better tailor treatments for STS patients. Future research should focus on the diverse histological subtypes of STS to fully understand and leverage the immunomodulatory effects of these therapies.

### Electronic supplementary material

Below is the link to the electronic supplementary material.

Supplementary Material 1

## Data Availability

No datasets were generated or analysed during the current study.

## Abbreviations

CP: Cyclophosphamide
DAPI: 4’,6-Diamidino-2-Phenylindole
DL: Dose Level
ECOG: Eastern Cooperative Oncology Group
FFPE: Formalin-Fixed Paraffin-Embedded
IHC: Immunohistochemistry
IF: Immunofluorescence
MTC: Metronomic Chemotherapy
OS: Overall Survival
PD-1: Programmed Death-1
PFS: Progression-Free Survival
RECIST: Response Evaluation Criteria in Solid Tumors
STS: Soft-Tissue Sarcomas
TME: Tumor Microenvironment
TSA: Tyramide System Amplification
