# Supplementary material for: Impact of metronomic trabectedin combined with low-dose cyclophosphamide on sarcoma microenvironment and correlation with clinical outcome: results from the TARMIC study
Source: Mol Cancer. 2024 Feb 19;23:37. doi: 10.1186/s12943-024-01942-y (PMC10875852; doi:10.1186/s12943-024-01942-y)
Supplement: Supplementary file 1 — Supplementary Material 1 [file 12943_2024_1942_MOESM1_ESM.doc]

**SUPPLEMENTARY TABLES AND FIGURES**

**Supplementary table 1. Antibodies used for the IHC and IF assays**

| **Antibody** | **References** | **Species** | **Clone** | **Source** | **Concentration (ug/mL)** | **Antigen Retrieval** | **Secondary Antibody** | **Tertiary Reagents** |
| --- | --- | --- | --- | --- | --- | --- | --- | --- |
| Immunohistochemistry | | | | | | | | |
| CD8 | M7103 | Mouse IgG1 | C8/144B | Agilent | 1.6 | pH9 | Polyview Plus HRP (anti-mouse) reagent | DAB |
| Immunofluorescence | | | | | | | | |
| CD68 | M0876 | Mouse IgG3 | PG-M1 | Agilent | 0.3 | pH6 | Polyview Plus HRP (anti-mouse) reagent | Alexa Fluor™ 647 Tyramide Reagent |
| CD163 | Mob460 | Mouse IgG1 | 10D6 | DBS | 0.6 | pH6 | Polyview Plus HRP (anti-mouse) reagent | Alexa Fluor™ 555 Tyramide Reagent |

| **Supplementary Table 2. Patient characteristics**  **Dose escalation part (n=20)** | |
| --- | --- |
| **Variable** |  |
| **Gender, n (%)** |  |
| Male | 10 (50.0) |
| Female | 10 (50.0) |
| **Age** |  |
| Median, years (range) | 61 (27–73) |
| **ECOG PS, n (%)** |  |
| 0 | 7 (35.0) |
| 1 | 13 (65.0) |
| **Histological subtype (%)​** |  |
| Leiomyosarcoma | 15 (5.0) |
| Undifferentiated pleiomorphic sarcoma | 2 (15.0)​ |
| Well-differentiated liposarcoma | 1 (40.0) |
| Myxoid liposarcoma | 1 (15.0) |
| Synovial sarcoma | 1 (5.0)​ |
| **Grade (%)** |  |
| 1 | 3 (5.0) |
| 2 | 7 (35.0) |
| 3 | 4 (20.0) |
| Unknown | 6 (30.0) |
| **Stage, n (%)** |  |
| Locally advanced | 1 |
| Metastatic | 19 |
| **Prior lines of chemotherapy in the advanced setting n (%)​** |  |
| 0 | 0 (0.0) |
| 1​ | 6 (70.0) |
| 2​ | 5 (30.0) |
| > 2​ | 9 (0.0) |

* ECOG PS, Eastern Cooperative Oncology Group performance status; UPS, undifferentiated pleomorphic sarcoma.

| **Supplementary Table 3. Patient characteristics**  **Phase 2 part (n=30)** | |
| --- | --- |
| **Variable** |  |
| **Gender, n (%)** |  |
| Male | 17 (50.0) |
| Female | 13 (50.0) |
| **Age** |  |
| Median, years (range) | 67 (23–80) |
| **ECOG PS, n (%)** |  |
| 0 | 7 (35.0) |
| 1 | 23 (65.0) |
| **Histological subtype (%)​** |  |
| Undifferentiated pleiomorphic sarcoma | 9 (15.0)​ |
| Leiomyosarcoma | 6 (5.0) |
| Synovial sarcoma | 5 (5.0)​ |
| Well-differentiated/dedifferentiated liposarcoma | 4 (40.0) |
| Myxoid /round cell liposarcoma | 2 (15.0) |
| Others | 4 (5.0)​ |
| **Grade (%)** |  |
| 2 | 13 (35.0) |
| 3 | 11 (20.0) |
| Unknown | 6 (30.0) |
| **Stage, n (%)** |  |
| Locally advanced | 7 |
| Metastatic | 23 |
| **Prior lines of chemotherapy in the advanced setting n (%)​** |  |
| 0 | 0 (0.0) |
| 1​ | 8 (70.0) |
| 2​ | 11 (30.0) |
| > 2​ | 11 (0.0) |

| Supplementary Table 4. Number of patients presenting at least one adverse event related to Trabectedin and/or Cyclophosphamide in the dose escalation phase (n=20) | | | | | | | | |
| --- | --- | --- | --- | --- | --- | --- | --- | --- |
|  | **Maximum intensity** | | | | | | | |
|  | **Grade 1** | | **Grade 2** | | **Grade 3** | | **Grade 4** | |
|  | **n** | **%** | **n** | **%** | **n** | **%** | **n** | **%** |
| **Blood lymphatic** |  |  |  |  |  |  |  |  |
| Anemia | 5 | 31.2 | 6 | 37.5 | . | . | . | . |
| **Gastrointestinal disorders** |  |  |  |  |  |  |  |  |
| Diarrhea | 2 | 12.5 | . | . | . | . | . | . |
| Gastrointestinal pain | 3 | 18.8 | . | . | . | . | . | . |
| Mucositis oral | 4 | 25 | 1 | 6.2 | . | . | . | . |
| Nausea | 8 | 50 | 4 | 25 | . | . | . | . |
| Vomiting | 4 | 25 | 1 | 6.2 | . | . | . | . |
| **General disorders** |  |  |  |  |  |  |  |  |
| Fatigue | 8 | 50 | 6 | 37.5 | 1 | 6.2 | . | . |
| **Laboratory Investigations** |  |  |  |  |  |  |  |  |
| Alanine aminotransferase increased | 2 | 12.5 | 1 | 6.2 | . | . | . | . |
| Alkaline phosphatase increased | . | . | 2 | 12.5 | . | . | . | . |
| Aspartate aminotransferase increased | 3 | 18.8 | . | . | . | . | . | . |
| CPK increased | 2 | 12.5 | 1 | 6.2 | . | . | 1 | 33.3 |
| GGT increased | . | . | . | . | 2 | 12.5 | 1 | 33.3 |
| Lymphopenia | 1 | 6.2 | 4 | 25 | 4 | 25 | . | . |
| Neutropenia | . | . | 2 | 12.5 | 3 | 18.8 | 1 | 33.3 |
| Thrombocytopenia | 2 | 12.5 | 3 | 18.8 | . | . | . | . |
| Leucopenia | . | . | 2 | 12.5 | 1 | 6.2 | . | . |
| **Metabolism and nutrition disorders** |  |  |  |  |  |  |  |  |
| Anorexia | 2 | 12.5 | . | . | . | . | . | . |
| **Nervous system disorders** |  |  |  |  |  |  |  |  |
| Dysgeusia | 2 | 12.5 | . | . | . | . | . | . |

| **Supplementary Table 5 Number of patients presenting at least one adverse event related to Trabectedin and/or Cyclophosphamide in the phase 2 part (n=30)** | | | | |
| --- | --- | --- | --- | --- |
|  | **Maximal intensity** | | | |
|  | **Grade 1-2** | | **Grade 3-4** | |
|  | **.** | **.** | **.** | **.** |
|  | **n** | **%** | **n** | **%** |
|  | 17 | 56.7 | 1 | 3.3 |
| **Blood lymphatic** |  |  |  |  |
| Anemia |  |  |  |  |
| **Gastrointestinal disorder** |  |  |  |  |
| Constipation | 4 | 13.3 | . | . |
| Mucositis oral | 7 | 23.3 | . | . |
| Nausea | 18 | 60.0 | . | . |
| Vomiting | 11 | 36.7 | . | . |
| **General disorders** |  |  |  |  |
| Fatigue | 24 | 80.0 | . | . |
| **Laboratory Investigations** |  |  |  |  |
| Alanine aminotransferase increased | 10 | 33.3 | 2 | 6.7 |
| Alkaline phosphatase increased | 5 | 16.7 | . | . |
| Aspartate aminotransferase increased | 9 | 30.0 | 1 | 3.3 |
| CPK increased | 6 | 20.0 | 1 | 3.3 |
| GGT increased | 4 | 13.3 | 4 | 13.3 |
| Lymphopenia | 11 | 36.7 | 15 | 50.0 |
| Neutropenia | 6 | 20.0 | 6 | 20.0 |
| Thrombocytopenia | 6 | 20.0 | 3 | 10.0 |
| **Metabolism and nutrition disorders** |  |  |  |  |
| Anorexia | 8 | 26.7 | . | . |
| **Nervous disorders** |  |  |  |  |
| Myalgia | 3 | 10.0 | . | . |
| Dysgeusia | 3 | 10.0 | . | . |

**
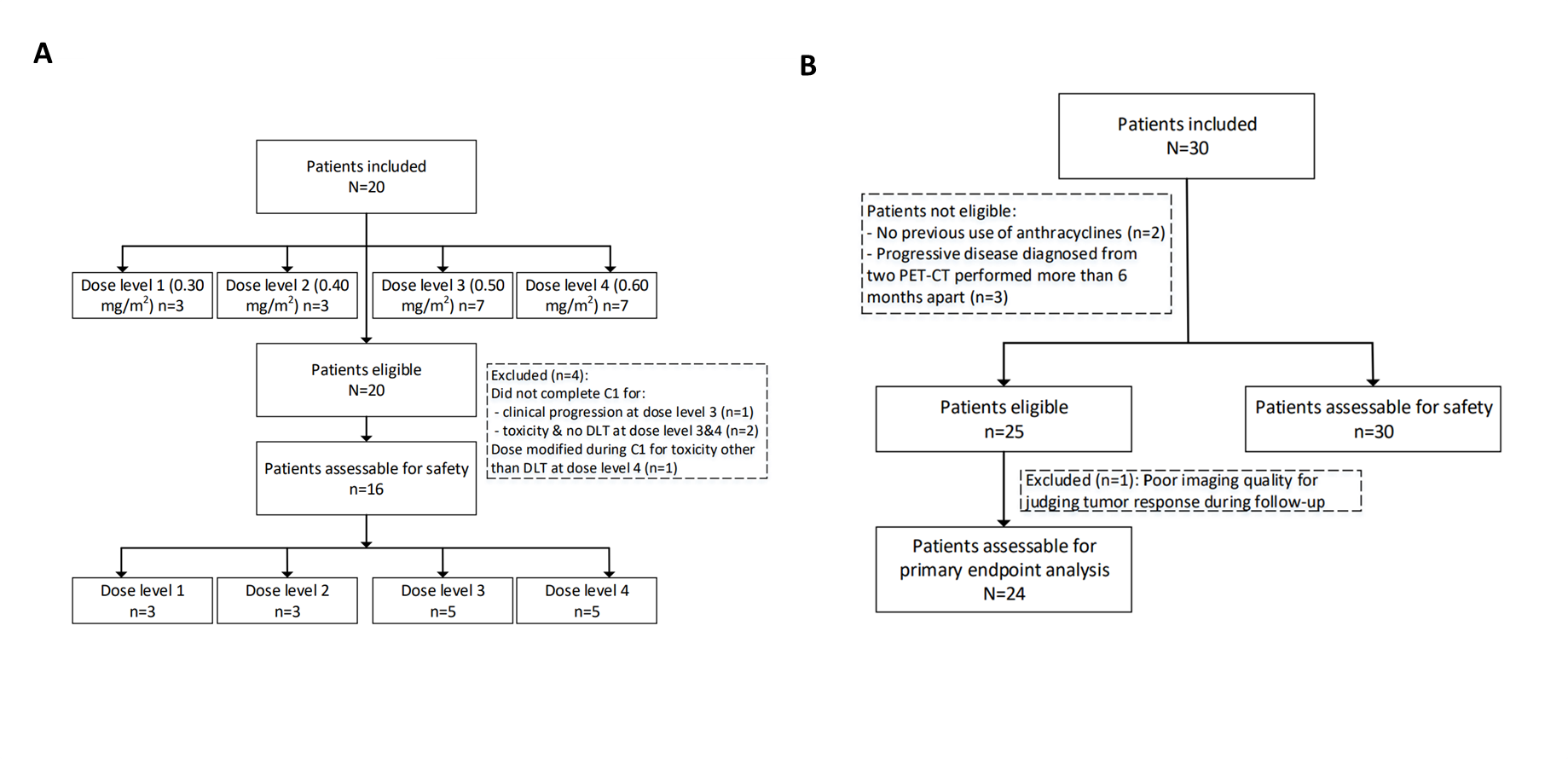
**

**Supplementary Figure 1**. Flow chart of the TARMIC study **A** Dose escalation part **B** Phase 2 part


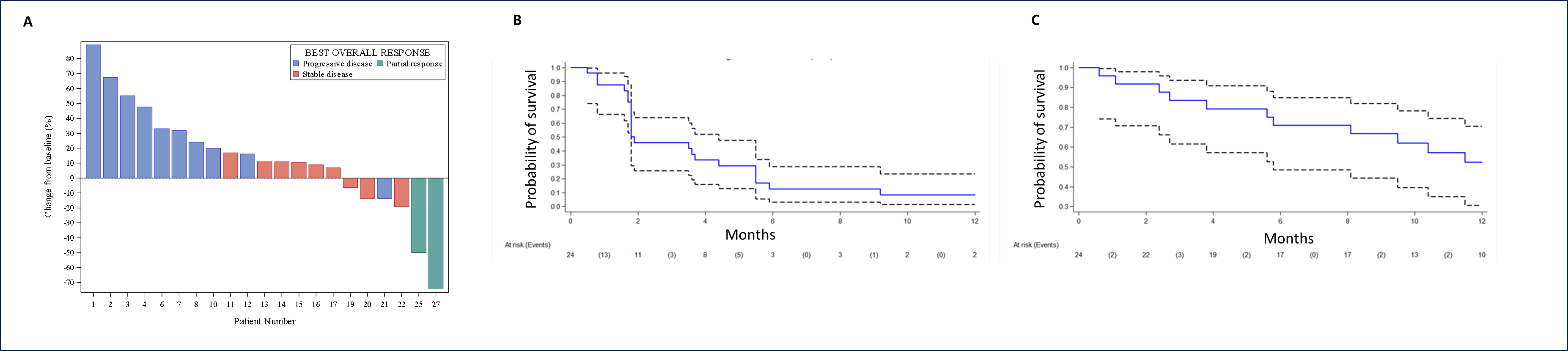


**Supplementary Figure 2**. Waterfall plots of tumor response **A** and Kaplan–Meier curves of progression-free **B** and overall survival **C** of patients enrolled in the phase 2 part and evaluable for efficacy (n=24). Only patients with available tumor assessments after central review at data cutoff are shown. Changes in tumor size were centrally assessed by blinded independent review according to Response Evaluation Criteria in Solid Tumors (RECIST) 1.1. Maximum change in sum of diameters from baseline is shown on the waterfall plots (**A**).


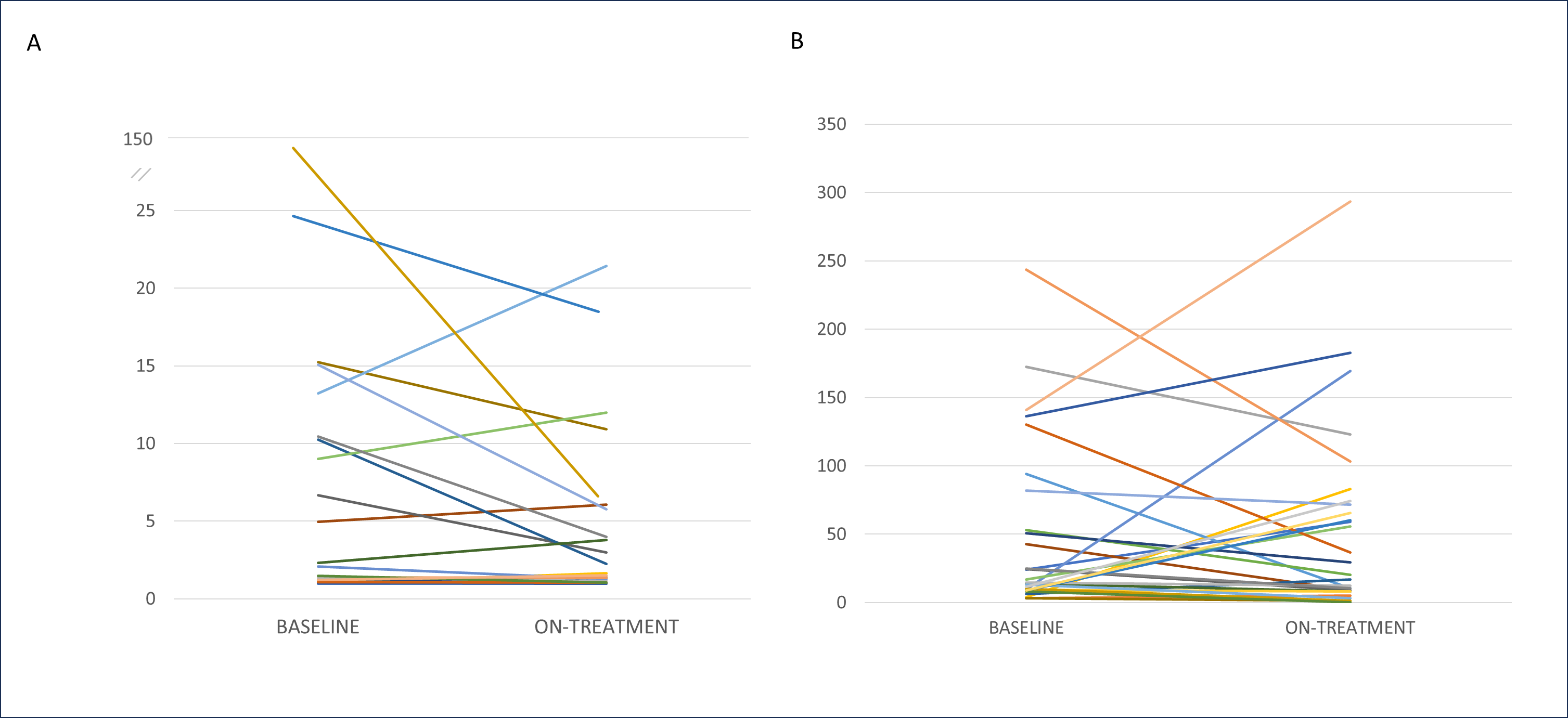


**Supplementary Figure 3**. Change in M2 macrophage (A) and CD8+ T cells density within individual pairs of tumors from pre- to on-treatment from patients enrolled in the TARMIC study (n=28)
